# Type 2 diabetes – unmet need, unresolved pathogenesis, mTORC1-centric paradigm

**DOI:** 10.1007/s11154-020-09545-w

**Published:** 2020-03-04

**Authors:** Jacob Bar-Tana

**Affiliations:** Hebrew University Medical School, 91120 Jerusalem, Israel

**Keywords:** Type 2 diabetes, Metabolic syndrome, Insulin resistance, Mammalian target of rapamycin (mTOR)

## Abstract

The current paradigm of type 2 diabetes (T2D) is gluco-centric, being exclusively categorized by glycemic characteristics. The gluco-centric paradigm views hyperglycemia as the primary target, being driven by resistance to insulin combined with progressive beta cells failure, and considers glycemic control its ultimate treatment goal. Most importantly, the gluco-centric paradigm considers the non-glycemic diseases associated with T2D, e.g., obesity, dyslipidemia, hypertension, macrovascular disease, microvascular disease and fatty liver as ‘risk factors’ and/or ‘outcomes’ and/or ‘comorbidities’, rather than primary inherent disease aspects of T2D. That is in spite of their high prevalence (60–90%) and major role in profiling T2D morbidity and mortality. Moreover, the gluco-centric paradigm fails to realize that the non-glycemic diseases of T2D are driven by insulin and, except for glycemic control, response to insulin in T2D is essentially the rule rather than the exception. Failure of the gluco-centric paradigm to offer an exhaustive unifying view of the glycemic and non-glycemic diseases of T2D may have contributed to T2D being still an unmet need. An mTORC1-centric paradigm maintains that hyperactive mTORC1 drives the glycemic and non-glycemic disease aspects of T2D. Hyperactive mTORC1 is proposed to act as double-edged agent, namely, to interfere with glycemic control by disrupting the insulin receptor-Akt transduction pathway, while concomitantly driving the non-glycemic diseases of T2D. The mTORC1-centric paradigm may offer a novel perspective for T2D in terms of pathogenesis, clinical focus and treatment strategy.

## Type 2 diabetes - unmet need

Type 2 Diabetes (T2D) consists of two stages, pre-diabetes and diabetes, with yearly conversion rate of 5–10%. The two stages are defined by their respective glycemic criteria based on HbA1C (5.7–6.4%; ≥6.5%), fasting plasma glucose (FPG) (100–125 mg/dL; ≥ 126 mg/dL) or oral glucose tolerance (OGT) (2 h plasma glucose 140–199 mg/dL; ≥ 200 mg/dL) [1]. T2D is epidemic, with average global diabetes prevalence of 8.8% of the world population aged 20–79 years, and expected to further increase to 9.9% by the year 2045 [2]. Diabetes prevalence in some countries may reach 30% of the local adult population. Prevalence estimates for pre-diabetes vary widely depending on the diagnostic test used, and amounts to >30% of world adult population [3].

Beyond its glycemic presentations, diabetes patients present a variety of highly prevalent non-glycemic diseases that drive T2D morbidity and mortality. Thus, close to 90% of diabetes adult patients are overweight or obese [4], resulting in the ‘diabesity’ connotation [5]. About 30–60% of Western diabetes patients are dyslipidemic (hypertriglyceridemia, small dense LDL-Cholesterol (sdLDL-C), low HDL-C) (6), reaching 60–90% prevalence in Asian population [7, 8]. Similarly, 60–85% of diabetes patients are hypertensive [9], and about 60% present non-alcoholic fatty liver disease (NAFLD) [10]. Moreover, diabetes patients have a significantly higher incidence of Alzheimer disease (AD) [11], and an increased incidence of a variety of cancers (liver, pancreas, colorectal, bladder, breast) [12]. Most importantly, about 30% of all diabetes patients present with ‘macrovascular’ (cardio- / cerebro- / peripheral-vascular) disease, being a major cause of morbidity, and accounting for half of all deaths [13]. Concomitantly, T2D patients are inflicted by a variety of ‘microvascular’ diseases, including diabetic nephropathy (30–50% prevalence [14]), retinopathy (30% prevalence [15]), and peripheral polyneuropathy (lifetime prevalence 30–50% [16]).

Of note, most of the non-glycemic diseases of T2D are already evident during the pre-diabetes stage of T2D, namely, prior to the appearance of solid hyperglycemia. Thus, most pre-diabetic patients are obese and/or hypertensive and/or dyslipidemic, being classified as non-diabetic Metabolic Syndrome patients [17]. Most importantly, pre-diabetes is already associated with an established cardiovascular disease [18,19], nephropathy [20], neuropathy [21,22], retinopathy [23] and all-cause mortality [24].

**Fig. 1:**
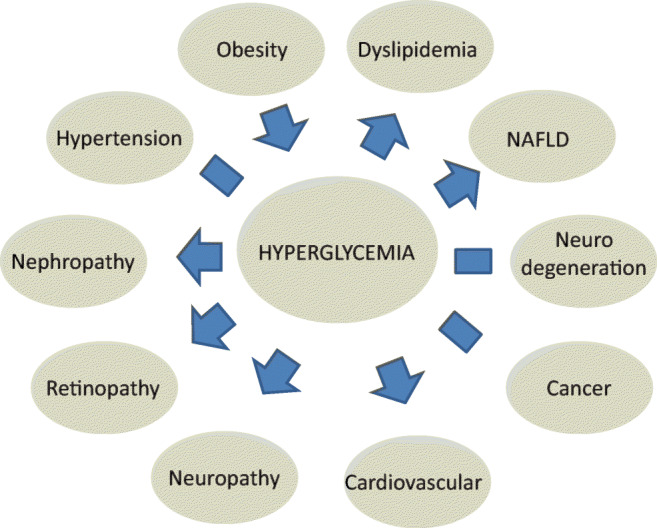
Gluco-centric paradigm of T2D. Hyperglycemia as T2D primary pathology. The other disease aspects of T2D are considered ‘risk factors’ and/or ‘outcomes’ and/or ‘comorbidities’

In spite of its phenotypic complexity, the current T2D paradigm is fully gluco-centric, being exclusively categorized by its glycemic characteristics. The gluco-centric view of T2D considers hyperglycemia as T2D primary pathology, and glycemic control its ultimate treatment. The other disease aspects of T2D are considered ‘risk factors’ (e.g., obesity) and/or ‘outcomes’ (macrovascular disease, microvascular disease, dyslipidemia, NAFLD) and/or ‘comorbidities’ (e.g., hypertension, cancer, neurodegeneration) (Fig. 1), rather than primary disease aspects of T2D. This gluco-centric view is shared by Diabetologists (1), Pharma, and Regulatory Authorities [25].

In line with the gluco-centric paradigm of T2D, pharmacological treatment focuses on glycemic control, usually initiated upon reaching the hyperglycemic diabetes stage of T2D [1]. Current antidiabetic drugs, except of metformin and thiazolidinediones, consist of agents that promote insulin secretion (sulphonylureas, GLP1 analogs, DPP4i), increase glucose excretion in urine (SGLT2i), or decrease dietary glucose absorption (acarbose), followed by insulin(s). However, in spite of the pharmacological efforts and economic burden [26] made in controlling hyperglycemia, about 40% of T2D patients still fail to reach glycemic control (A1C < 7%) [27–29]. Moreover, failure is more common in patients prescribed insulin(s), whereby 65% of patients fail to reach glycemic targets [27]. Most importantly, except for pioglitazone (having a limited use due to side effects) and GLP1 analogs, no drug has proved effective in preventing / delaying the progressive failure of beta cells in T2D patients.

Of note, hyperglycemia levels in T2D patients within the 7–10% HbA1C range are positively associated with risk to develop the macrovascular and/or microvascular diseases of T2D [30]. However, the macrovascular disease is unaffected [31–34] while the nephropathy disease is only mildly affected [35] by anti-diabetic drugs which target hyperglycemia (HbA1C < 7.0%), implying dubious causal association between hyperglycemia levels and the macro- and micro-vascular diseases of T2D. More surprisingly, intensive glycemic control (HbA1C 6.4% vs 7.5%), accomplished by more use of insulin(s) and oral drug combinations is reported to be associated with increased mortality [31], in particular in T2D patients presenting with diabetic nephropathy [36].

Within the framework of cardiovascular outcome trials (CVOT) required by the FDA since 2008 for proving safety of new anti-diabetic drugs, some GLP1 analogs and SGLT2i proved statistical cardiovascular benefit over placebo in T2D patients with cardiovascular disease [37–39]. In light of their mild glucose lowering efficacy (decrease in HbA1C of 0.24–0.58%), their cardiovascular benefit remains to be investigated. Most importantly, the number of patients who need to be treated (NNT) for a period of 3–4 years to prevent one cardiovascular MACE outcome by Empagliflozin, Canagliflozin or Liraglutide amounts to 62, 22 and 53, respectively (adapted from ref. 37-39). Similarly, the respective NNT to prevent a hospitalization for heart failure amounts to 71, 31, and 166 (adapted from ref. 37-39), implying a limited efficacy in alleviating the macrovascular disease of T2D patients.

In light of failure to adequately target the macrovascular disease of T2D by hypoglycemic measures, standard of care (SOC) treatments are presently directed to cardiovascular risk factors known to precipitate and drive the macrovascular disease independently of the glycemic context (1). In line with that, T2D patients are routinely prescribed with 1–3 hypolipidemic agents (high-intensity statin, ezetimibe, fibrates, anti-PCSK9 antibody [40]), aspirin, and 1–3 hypotensive drugs (beta-blocker, thiazide, Ca-channel blocker, ACEi, angiotensin receptor blocker (ARB)), in addition to 1–6 hypoglycemic agents (metformin, sulphonylurea, DPP4i, SGLT2i, GLP1 analog, acarbose, insulin(s)) [1]. That is in addition to dietary and exercise recommendations [1]. However, the recommended SOC is only partially productive in delaying the diabetes stage of pre-diabetes patients [41] or in alleviating the macrovascular disease of T2D patients [42]. Also, the success in targeting concomitantly the glycemic, dyslipidemic and hypertensive diseases of T2D is less than 25% [28, 29]. Also, some of the drugs designed to treat the non-glycemic aspects of T2D (e.g., beta-blockers, thiazides, statins) may counteract glycemic control [43–45]. Most importantly, in spite of the enormous medical efforts and economic burden invested in treating T2D, the disease is still progressive resulting in unmet suffering, morbidity and premature death.

## Type 2 diabetes - unresolved pathogenesis

The current pathogenic paradigm for T2D maintains that glycemic control reflects the interplay between insulin availability and the sensitivity / resistance to insulin of the main tissues engaged in carbo-lipid metabolism, namely, liver, muscle and adipose tissue. Insulin availability is mainly determined by pancreatic beta cells capacity, being modulated by the degradation of circulating insulin in liver. Insulin promotes hepatic and muscle glycogenesis, suppresses hepatic gluconeogenesis, drives glucose uptake and its utilization in muscle (mainly) and adipose tissue, and suppresses adipose lipolysis. Hence, peripheral resistance to insulin implies unrestrained hepatic glucose production, and suppression of muscle and adipose glucose uptake, resulting in hyperglycemia. Peripheral resistance to insulin further implies unrestrained adipose lipolysis, resulting in efflux of long-chain fatty acids (LCFA) that may affect liver and muscle carbo-lipid metabolism [46].

**Fig. 2:**
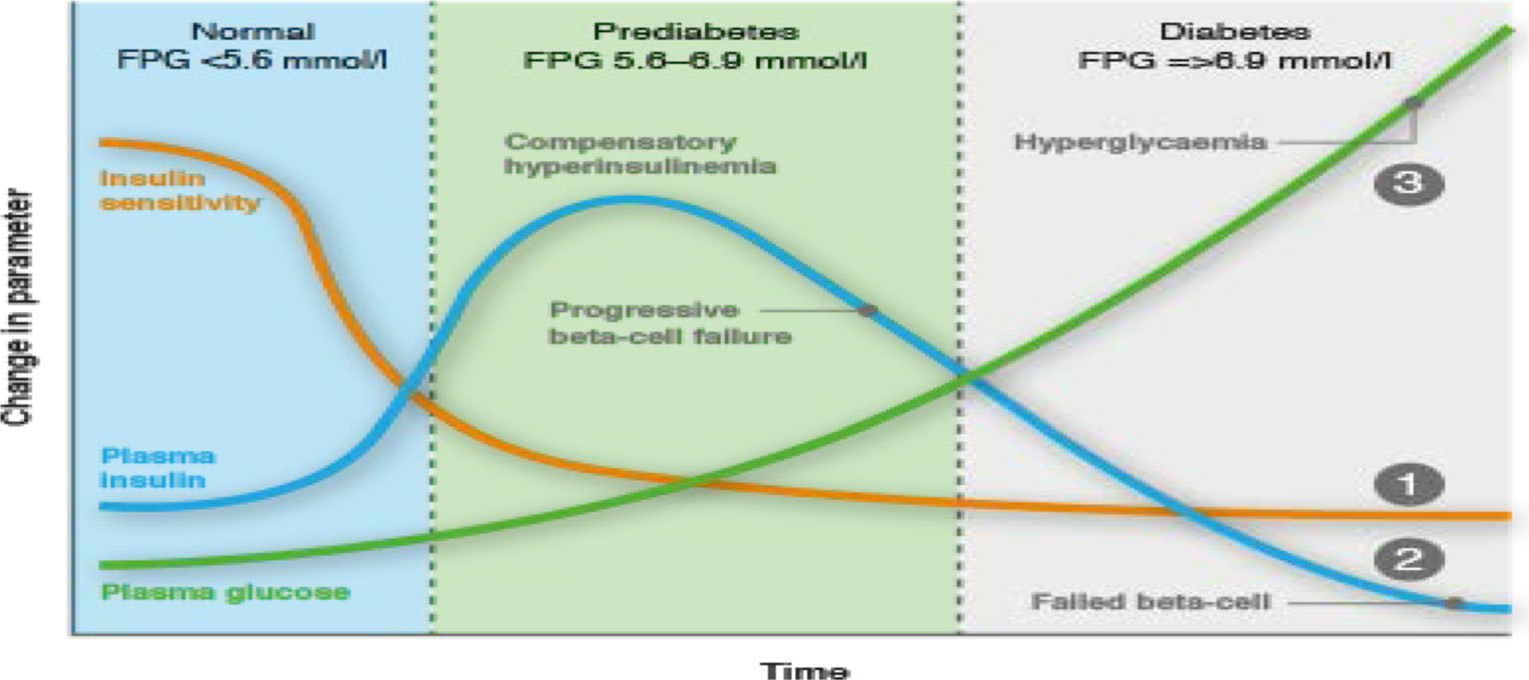
Pre-diabetes / Diabetes paradigm of T2D. Peripheral resistance to insulin in the pre-diabetes stage is counterbalanced by increased insulin secretion by beta cells, resulting in normoglycemia or mild hyperglycemia. Peripheral resistance to insulin in the diabetes stage results in progressive hyperglycemia due to progressive beta cells failure

The interplay between pancreatic insulin production and peripheral resistance to insulin defines the current pathogenic paradigm of the pre-diabetes and diabetes stages of T2D (Fig. 2). The pre-diabetes stage is considered to reflect progressive peripheral resistance to insulin, being counterbalanced by increased insulin secretion by beta cells. The resultant hyperinsulinemia offsets the peripheral resistance, resulting in maintaining fasting plasma glucose levels within the <126 mg/dL range. The diabetes stage that follows reflects failure / insufficiency of beta cells to compensate for the prevailing peripheral resistance to insulin, resulting in progressive hyperglycemia [47].

The current pathogenic paradigm for T2D leaves unanswered the following questions-.

### Pre-diabetes insulin resistance vs. hyperinsulinemia: Which comes first?

The classical pathophysiology paradigm of the pre-diabetes stage of T2D maintains that peripheral insulin resistance comes first, followed by compensatory hyperinsulinemia due to increase in beta cells mass and activity [48]. Indeed, peripheral insulin resistance has been verified in normoglycemic first-degree relatives of diabetes parents, having a very high life-time risk of developing diabetes [49]. However, these individuals present as well with fasting hyperinsulinemia, making it impossible to decide between insulin resistance and hyperactive beta cells as primary driver. Also, claiming resistance as the primary driver calls for a humoral and/or neuronal agent(s) that may mediate between peripheral resistance and beta cells mass and function during the early normoglycemic normolipemic pre-diabetes stage. However, in spite of repeated efforts, no such mediators have yet been definitively identified.

Alternatively, one may argue for beta cells hyperactivity as a primary driver, at least under conditions of nutrient excess, resulting in primary hyperinsulinemia followed by downregulation of peripheral insulin receptors and/or their signaling pathway [50]. Indeed, acute or chronic increase in plasma insulin in healthy normoglycemic subjects results in significant decrease in insulin-stimulated glucose disposal [51, 52], implying that primary hyperinsulinemia may drive insulin resistance. Also, a variety of high-fat diet (HFD) feeding studies in rats and mice demonstrate fasting hyperinsulinemia at the earliest feeding stage prior to any detectable increase in plasma glucose as a surrogate for insulin resistance (50). However, downregulation of insulin receptors by 90% may still allow for adequate insulin signaling due to spare insulin receptors [53], implying that peripheral insulin resistance must further involve post receptor defects [54, 55]. However, no agents have yet been identified which may drive post receptor defects due to hyperinsulinemia. Hence, the question of which comes first in driving the pathogenesis of the pre-diabetes stage of T2D still remains unresolved.

### Selective insulin resistance: The insulin paradox

**Fig. 3:**
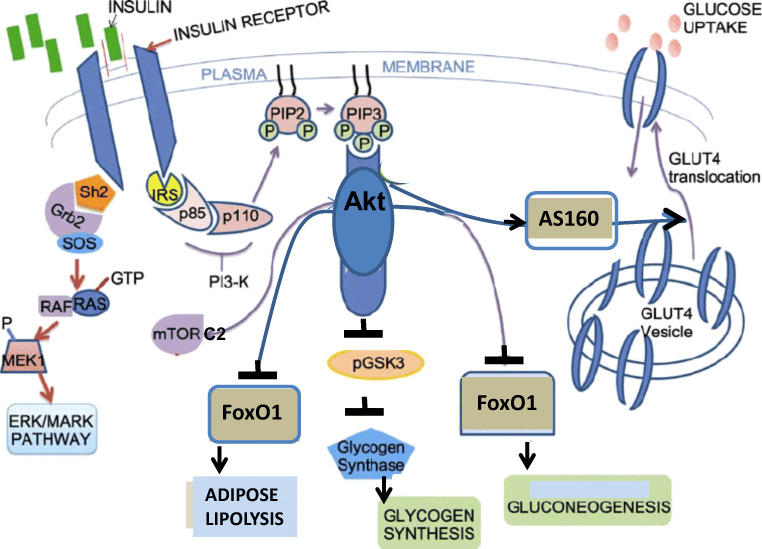
IR-Akt transduction pathway. Glycemic control mediated by the IR-Akt transduction pathway

Insulin binding to the insulin receptor (IR) results in forming an IR / insulin receptor substrate (IRS) node attached to the plasma membrane. Phosphorylation of IRS tyrosines by the IR tyrosine kinase results in binding sites for the PI3K and its activation, followed by phosphorylating phosphatidylinositol 4,5-bisphosphate (PIP2) to yield the membrane bound phosphatidylinositol 3,4,5-triphosphate (PIP3). PIP3 binds and activates PDK1 which phosphorylates Akt/PKB(Thr308). Further phosphorylation of Akt/PKB(Ser473) by PIP3-activated mTORC2 results in the fully activated phospho-Akt(Thr308, Ser473). Signal termination is achieved by the PIP3 phosphatase PTEN or the protein phosphatases PP2A and PHLPP which dephosphorylate Akt(Thr308) and Akt(Ser473), respectively. The IR-Akt transduction pathway controls carbo-lipid metabolism in response to insulin (Fig. 3). Thus, phosphorylation of glycogen synthase kinase 3 (GSK3) by Akt results in its suppression and in activation of liver and muscle glycogen synthase; phosphorylation of AS160/TBC1D4 by Akt results in muscle and adipose glucose uptake by translocating GLUT4 to the plasma membrane; and phosphorylation of the transcription factor FOXO1(Thr24,Ser256) by Akt results in its cytosolic sequestration and transcriptional suppression of hepatic gluconeogenesis and adipose fat lipolysis. Hence disruption of the IR-Akt transduction pathway resists insulin action and results in unrestrained hyperglycemia and adipose lipolysis [56].

In addition to the IR/IRS node that drives Akt activation, binding of insulin to IR results in generating the IR/Shc/Grb2/SOS node that drives the activation of the Ras/RAF/MEK/Erk1,2/p90RSK transduction pathway. However, Erk1,2/p90RSK are considered to transduce mitogenic signaling, in contrast to Akt/PKB which controls carbo-lipid metabolism (Fig. 3).

Whereas the IR-Akt transduction pathway may account for insulin resistance in the glycemic context of T2D, other disease aspects of T2D surprisingly present full response to insulin, implying “selective insulin resistance” [57]. The first example of an “insulin paradox” was concerned with hepatic lipogenesis, namely, de novo fatty acids biosynthesis followed by their esterification to yield lipids. Lipogenesis partially accounts for the NAFLD disease of T2D, and proved to be fully responsive to insulin in T2D animal models and patients, in face of resistance to insulin in the glycemic context [57]. Another example has been realized in studying VLDL production in human subjects or animal models. Since insulin suppresses VLDL production [58], IR knockout predicts dyslipidemia. However, IR knockout results in hyperglycemia as expected, but the dyslipidemia of T2D is avoided [59, 60], implying an apparent selective role of the IR in controlling hyperglycemia as contrasted with dyslipidemia.

Several explanations were offered to solve the gluconeogenesis / lipogenesis insulin paradox. These have argued for insulin-independent lipid synthesis [61, 62], or hepatic / adipose selective sensitivity to insulin [63, 64], or extrahepatic insulin effects (hypothalamic, adipose, pancreatic alpha-cell) [65], or selective / differential activity of Akt in phosphorylating its downstream substrates [66], or hepatic zone specificity of carbo-lipid metabolism [67]. However, all were limited to the glycemic-steatosis paradox, while failing to realize that, except of glycemic control, response to insulin is essentially the rule in T2D, rather than the exception. Indeed, the diabesity of T2D reflects insulin-responsive body weight gain, in face of resistance to insulin in the glycemic and lipolysis context [68–70]. Also, the hypertension disease of T2D reflects insulin-responsive sympathetic activity [71, 72], renal sodium reabsorption [73] and endothelial vasoconstriction [74]. Similarly, T2D hyperuricemia reflects suppression by insulin of renal uric acid clearance [75]. Also, the macrovascular disease of T2D reflects insulin-responsive dyslipidemia [59], hypertension [70–74], diabetic cardiomyopathy [76], vascular smooth muscle cells (VSMC) proliferation, endothelial dysfunction and prothrombosis [77]. Of note, the same tissue may present resistance to insulin in the glycemic context together with response to insulin in a non-glycemic aspect. Hence, the interplay between response and resistance to insulin in shaping the pathogenesis of T2D still remains unresolved, calling for an insulin-dependent, context-dependent, resistance-response unifying paradigm.

### Insulin resistance and mitochondrial dysfunction: The athlete paradox and the insulin sensitizers riddle

Insulin resistance has been proposed to be driven by ‘mitochondrial dysfunction’ with decrease in mitochondria content, size, biogenesis, electron flux and oxidative phosphorylation, and increased oxidative stress [78, 79]. Indeed, muscle insulin resistance with concomitant mitochondrial dysfunction has been verified in diabetes patients, obese prediabetic subjects, insulin-resistant lean non-diabetic off-springs of T2D parents, insulin-resistant non-diabetic elderly and T2D animal models, implying a putative causal relationship [62]. Mitochondrial dysfunction has been proposed to result in suppression of fatty acid oxidation, followed by their esterification into intra-hepatic and intra-myocellular lipids (IMCL) [80]. Intracellular diacylglycerols and oxidative stress are proposed to activate novel PKCs (nPKC) and/or JNK respectively, resulting in serine/threonine phosphorylation of IRS1. Serine/threonine phosphorylation of IRS1 suppresses IRS tyrosine phosphorylation by the IR tyrosine kinase, resulting in disruption of the IR-Akt transduction pathway [62]. Indeed, hepatic steatosis is associated with insulin resistance [81], whereas increased beta-oxidation results in hepatic sensitivity to insulin [82]. However, the IMCL narrative still leaves unresolved the athlete paradox, whereby aerobic exercise is reported to result in increase in IMCL while counteracting muscle insulin resistance [83]. Moreover, the mitochondrial dysfunction narrative still leaves unresolved the insulin sensitizers’ riddle, whereby anti-diabetic agents that promote sensitization to insulin e.g., biguanides / metformin, thiazolidinedioones / pioglitazone, high-dose aspirin, berberine and others, are all reported to act as mitochondrial complex I inhibitors [84], implying that ‘mitochondrial dysfunction’ is more of a solution rather than a problem in the T2D context [85]. Also, mitochondrial ROS is reported by some (but refuted by others) to enhance insulin activity and/or counteract resistance to insulin, rather than exacerbate resistance due to putative oxidative stress [86–89]. Hence, the upstream driver(s) and respective downstream targets that drive insulin resistance by disrupting the IR-Akt transduction pathway still remain unresolved.

### Hyperactive beta cells vs beta cells failure: The beta cells dilemma

T2D is considered to follow a two-stage pre-diabetes / diabetes sequence, affixed by a demarcating plasma glucose value of 126 mg/dL, with annual conversion rate of 5–10% [1, 47]. The pre-diabetes stage is characterized by an increase in beta cells mass and activity, resulting in hyperinsulinemia which offsets peripheral insulin resistance. The pre-diabetes stage is proposed to be followed by progressive beta cells failure and an established diabetes status [1] (Fig. 2), ascribed to low innate beta cells mass [90] and/or stunned beta cells [91]. Beta cells failure may culminate in de-differentiation, mitochondrial dysfunction, ROS production, unfolded protein response (UPR) / endoplasmic reticulum (ER) stress and islet amyloid polypeptide (IAPP) overexpression, followed by glucolipotoxicity and apoptosis [92, 93]. The two-stage paradigm implies an apparent turning point in T2D pathogenesis and treatment policy. However, concomitantly with beta cells hyperactivity, the pre-diabetes stage presents loss of the first-phase of insulin secretion [94] and about 50% loss of beta cell number due to apoptosis [95, 96]. Hence, the pre-diabetes stage presents functional-structural failures which are carried over to, and progress during the hyperglycemic diabetes stage. Thus, similar to peripheral insulin resistance which prevails throughout the course of T2D, beta cells dysfunction and loss progress throughout the pre-diabetes / diabetes continuum, calling for a pathogenic paradigm that may account for beta cells concomitant hyperactivity and failure.

## Type 2 diabetes – mTORC1-centric unifying paradigm

In spite of the advances made in understanding and management of T2D, the disease is progressive, bearing high suffering, morbidity and mortality. Failure to realize the exhaustive pathogenic context of T2D may have contributed to T2D being still an unmet need. The current status calls for a unifying paradigm pointing to an upstream defined driver that may generate the multiple disease aspects of T2D, and which may offer an etiology-based target for an exhaustive treatment approach. The paradigm proposed below has been inspired by previous inputs made by David Sabatini [97], Mikhail Blagosklonny [98] and others.

### mTORC1 [97, 99]

The Mammalian target of rapamycin complex I (mTORC1) controls growth and metabolism in response to nutrients, energy and redox status. The mTORC1 complex consists of the mTOR kinase, the core subunits Raptor and mLST8, and the PRAS40 and DEPTOR inhibitory subunits. mTORC1 activation is enabled by two converging arms, namely, mTORC1 attraction to the lysosomal surface, and its activation by the constitutively-attached lysosomal Rheb. These two activation arms are independent, and each is affected by specific upstream effectors.

**Fig. 4:**
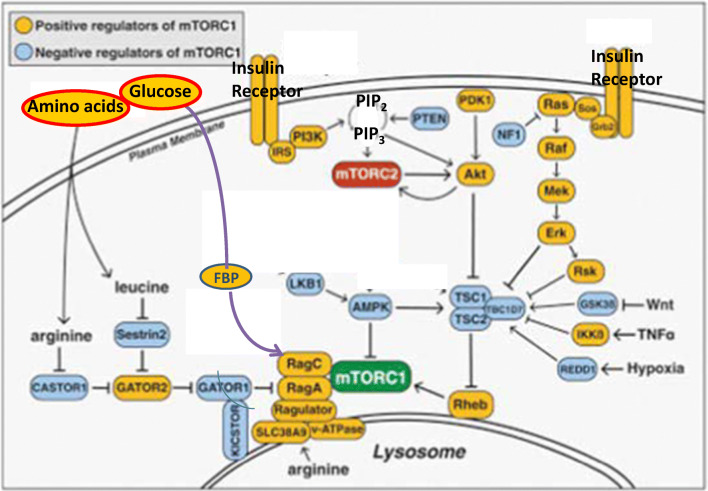
mTORC1. Binding of mTORC1 to the lysosomal surface is activated by glucose and/or amino acids, being mediated by lysosomal Rag.GTPase heterodimers. Lysosomal mTORC1 is activated by lysosomal Rheb.GTP, being activated by insulin via the IR-Akt and/or the IR-Erk/RSK transduction pathways. FBP - Fructose bi-phosphate

Binding of mTORC1 to the lysosomal surface via its Raptor subunit is mediated by the lysosomal Rag.GTPase heterodimers, RagA/B and RagC/D. Binding of mTORC1 requires the RagA/B.GTP and RagC/D.GDP conformation, being determined by lysosomal Ragulator, acting as specific GTP exchange factor (GEF) of Rags, and by lysosomal Gator1 and Folliculin-FNIP2 which function as GTPase activation proteins (GAP) for RagA/B and RagC/D, respectively. The Rag arm is affected by a variety of cytosolic and intra-lysosomal amino acids (e.g., leucine, arginine, glutamine) which bind to specific amino acid receptors (e.g., Sestrin, Castor1, SLC38A9) that interact, directly or indirectly, with the GAPs and/or GEFs that determine Rags conformation. Of importance, the Rag arm may also be activated by glucose [100]. Activation by glucose is mediated by binding of glucose-derived aldolase-bound fructose 1,6-bisphosphate (FBP) to Ragulator, resulting in RagA/B.GTP and its association with mTORC1 [101, 102]. Hence, the Rag arm mediates mTORC1 activation by amino acids and/or glucose, while suppressing mTORC1 activity in their absence (Fig. 4).

Once attracted to the lysosome, mTORC1 may be activated by lysosomal Rheb.GTP. Rheb may cycle between its active GTP and inactive GDP forms. The GTPase activity of Rheb is modulated by the Tuberous sclerosis complex (TSC), whereby its TSC2 subunit acts as a specific GAP of Rheb. The Rheb arm is activated upon suppressing TSC GAP and/or by suppressing TSC lysosomal attachment. TSC activity may be inhibited by specific phosphorylation of TSC2(Ser939,981,1130,1132,Thr1462) by Akt, resulting in a canonical transduction pathway that leads from insulin/IR to Rheb.GTP and mTORC1 activation [103]. mTORC1 activation by Akt is further complemented by phosphorylation of PRAS40(Thr246) by Akt, resulting in de-repressing mTOR [104]. Most importantly, TSC activity may similarly be inhibited by specific phosphorylation of TSC2(Ser540,644,1798) by activated Erk1,2 and/or p90RSK [105], resulting in an alternative transduction pathway that leads from insulin/IR to Rheb.GTP and mTORC1 activation. The two alternative pathways imply a functional redundancy of the Akt and the Erk1,2/RSK effectors in mediating mTORC1 activation by insulin/IR as well as by other growth factors / RTKs. Also, TSC activity may further be inhibited by specific phosphorylation of TSC2(Ser487,511) by activated IkapaB kinase (IKK) [106], resulting in mTORC1 activation by pro-inflammatory TNFa/TNFR, IL1/IL1R and LPS/TLR4. In contrast, TSC GAP may be activated by specific phosphorylation of TSC2(Ser1345,Thr1227) by AMPK / GSK3beta [107], resulting in suppressing mTORC1 activity by metabolic stress. Suppression of mTORC1 activity by AMPK is further complemented by phosphorylation of Raptor(Ser792) by AMPK [108], resulting in disrupting mTORC1 composition. Similarly to AMPK, multiple different metabolic stresses e.g., hypoxic, deoxy glucose, hyperosmotic, pH, may promote lysosomal TSC2 and/or increase its stability, resulting in inhibition of mTORC1 activity [109, 110]. Hence, mTORC1 activation is enabled by two converging hits, its lysosomal binding driven by nutrients (e.g., glucose, amino acids), and its activation by lysosomal Rheb.GTP driven by growth factors (e.g., insulin) (Fig. 4). In line with that, TSC1,2 knockout and constitutive RagA.GTP result in constitutive activation of mTORC1 under fasting conditions.

mTORC1 controls growth and metabolism by phosphorylating and/or affecting its downstream targets S6K1, 4EBP, CRTC2, lipin, ATF4, HIF1a, PPARg, PPARa, ULK1, TFEB and others [97, 99]. Phosphorylation of S6K1 and 4EBP results in ribosome biogenesis and in initiating CAP-dependent mRNA translation. Phosphorylation of CRTC2 and lipin results in activating the transcription factor SREBP and in driving lipogenesis and lipid synthesis. Phosphorylation of ATF4 and S6K1 results in purine and pyrimidine biosynthesis, respectively. Activation of HIF1a and SREBP transcription factors results in enhancing glycolysis and the pentose shunt. PPARg activation by mTORC1 promotes adipogenesis, while PPARa inhibition by mTORC1 suppresses fatty acid oxidation and ketogenesis. mTORC1 controls G1/S transition and G2/M progression. Most importantly, phosphorylation of the ULK1 kinase, Atg13 and the TFEB transcription factor by mTORC1 blocks autophagy and lysosome biogenesis. Not all immediate downstream targets of mTORC1 have presently been verified. However, downstream targets of mTORC1 may be inferred by their response to mTORC1 inhibitors (below).

Some mTORC1 activities in the T2D context are transduced by interacting with mTORC2. The mTORC2 complex consists of the mTOR kinase, mLST8, Rictor (instead of mTORC1 Raptor) mSin1, Deptor and Protor [97, 99]. mTORC2 phosphorylates and activates Akt(Ser473), the AGC protein kinases (PKA, PKG, PKC) and Serum and glucocorticoid kinase (SGK). mTORC2 activity is inhibited by phosphorylation of its mSin1 and Rictor subunits by S6K1, thus forming a negative feedback loop whereby activation of mTORC1 by Akt results in inhibition of Akt due to suppression mTORC2 activity by S6K1.

mTORC1 activity may be blocked by rapamycin and respective rapalogs. Rapamycin forms a complex with the FK506-binding protein (FKBP12), and the complex binds to the FRB domain of mTOR, resulting in allosteric inhibition of mTORC1. Hence, response to rapamycin may imply an apparent involvement of mTORC1. However, chronic exposure to rapamycin may also inhibit mTORC2, resulting in a false positive inference of mTORC1 involvement [111]. Also, not all mTORC1 downstream targets are affected by rapamycin (e.g., 4EBP), and lack of response to rapamycin may result in a false negative inference of mTORC1 involvement [112]. mTORC1 and mTORC2 may both be inhibited by mTOR kinase inhibitors (e.g., Torin).

### mTORC1-centric paradigm of T2D

mTORC1 activity may present two pathological extremes, namely, lack-of-function due to mutation of one of its core subunits, and gain-of-function due to genetic constitutive activation of its main drivers (e.g. RagA/B.GTP, Rheb.GTP). In between the two respective mutational extremes, wildtype mTORC1 activity may range between less- and hyper- active kinase, as function of metabolic stress and energy excess, respectively. *T2D is proposed to be primarily driven by chronic whole body hyper activation of mTORC1, induced by nutrients / energy excess / metabolites which concomitantly activate the RagA/B.GTP and the Rheb.GTP drivers of mTORC1. In line with that, T2D may be alleviated by suppressing mTORC1 hyper activation.* Specifically, mTORC1 hyper activation may be driven by chronic dietary carbohydrate excess of high glycemic index, resulting in concomitant activation of the glucose-induced RagA/B.GTP and the insulin-induced Rheb.GTP drivers of mTORC1. Hyper activation of mTORC1 may similarly be driven by chronic dietary excess of proteins rich in leucine and arginine. These amino acids may stimulate insulin secretion, resulting concomitantly in amino acid-induced RagA/B.GTP and insulin-induced Rheb.GTP. In line with that, caloric restriction, in particular carbohydrate restriction, may inhibit mTORC1 activity by repressing the Rag and Rheb arms due to nutrient and insulin restriction, respectively. Of note, modulation of mTORC1 activity by nutrients / energy excess / metabolites may further be affected by genetic and/or epigenetic and/or tissue and/or context-dependent factors that may determine the sensitivity of the Rag and Rheb arms to respective environmental / metabolic / nutrient conditions. Also, primary metabolic effects due to hyperactive mTORC1 may further be modulated by downstream secondary outcomes.

### Glycemic context of T2D. Resistance to insulin

**Fig. 5:**
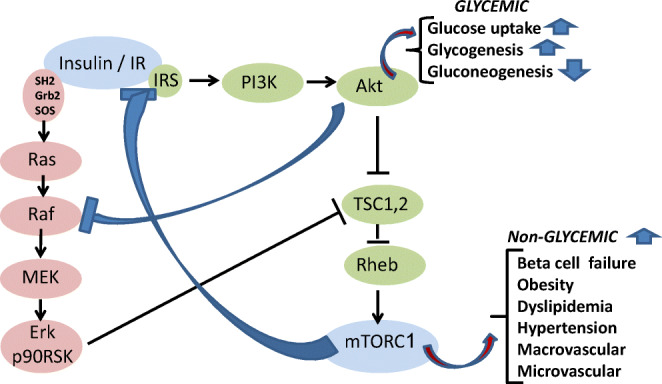
Resistance and response to insulin by mTORC1. Hyperactive mTORC1 inhibits the IR-Akt transduction pathway resulting in resistance to insulin and deranged glycemic control. Concomitantly, insulin-induced hyperactivation of mTORC1 by the IR-Erk/RSK transduction pathway drives the non-glycemic diseases of T2D. Inhibition of the IR-Akt transduction pathway by hyperactive mTORC1 results in activating the IR-Erk/RSK pathway and in mTORC1 hyper activation

Peripheral resistance to insulin in the glycemic context is proposed to be driven by disruption of the IR-Akt transduction pathway by hyperactive mTORC1 and its downstream S6K1 in liver, muscle and adipose tissue, namely, the main organs that control glucose production and its utilization. Thus, phosphorylation of IRS1(Ser307, 1101) by hyperactive S6K1, and phosphorylation of IRS1(Ser636/639, 422) by hyperactive mTORC1, result in suppressing IRS tyrosines phosphorylation by the IR tyrosine kinase, followed by IRS ubiquitination and degradation [113–115]. Also, phosphorylation of GRB10 by hyperactive mTORC1 results in disrupting IR/IRS by phospho-GRB10 [116, 117]. The IR-Akt transduction pathway is further disrupted by inhibition of Akt(Ser473) phosphorylation by mTORC2, due to suppression of mTORC2 kinase activity by hyperactive S6K1 [118, 119] (Fig. 5). Disruption of the IR-Akt pathway by hyperactive mTORC1/S6K1 results in liver and muscle glycogenolysis, liver gluconeogenesis, GLUT4 sequestration and unrestrained hyperglycemia. In line with that, genetic deletion of S6K1 protects mice from HFD-induced diabetes [120, 121]. Hence, resistance to insulin in the glycemic context is proposed to be congruent with mTORC1/S6K1 hyper activation.

### Non-glycemic context of T2D. Response to insulin

Disruption of the IR-Akt transduction pathway by hyperactive mTORC1 may still allow for sustained hyper activation of mTORC1 by insulin, being mediated by the IR/Ras/Raf/MEK/Erk/p90RSK/TSC/Rheb/mTORC1 transduction pathway, implying redundancy of IR-Akt and IR-Erk/RSK in activating mTORC1 [122, 123] (Figs. 4, 5). Moreover, the reciprocal relationship between the IR-Akt and the IR-Erk/RSK pathways, due to inhibitory phosphorylation of Raf(Ser259) by activated Akt [124, 125], implies enhancement of the IR-Erk/RSK activity upon inhibiting the IR-Akt pathway by hyperactive mTORC1. Hence, in face of resistance to insulin in the glycemic context, insulin-driven Erk/RSK may transduce a variety of mTORC1-mediated disease aspects of T2D (e.g., beta cell failure, obesity, NAFLD, dyslipidemia, hypertension, diabetes macro- and micro-vascular disease) as outlined below (Fig. 5). Indeed, IR knockout results in hyperglycemia, but also in protecting from non-glycemic diseases of T2D [59], implying an obligatory role for insulin and IR in driving the non-glycemic diseases of T2D.

#### Progressive beta cells failure

The IR-Erk/RSK transduction pathway is fully active in beta cells [126, 127], allowing for mTORC1 hyper activation in beta cells in response to nutrient excess, independently of the IR-Akt transduction pathway. Thus, nutrient excess is proposed to account for both, peripheral resistance to insulin in the glycemic context due to suppression of the IR-Akt transduction pathway by hyper active mTORC1 in liver, muscle and adipose tissue (III3), with concomitant increase in insulin production due to IR-Erk/RSK-induced mTORC1 hyper activation in beta cells. Of note, the concomitant hyper activation of mTORC1 in beta cells, liver, muscle and adipose tissue, makes redundant the question of which precedes which, and the search for respective mediators that may generate the insulin resistance / hyperinsulinemia phenotype during the pre-diabetes phase of T2D (Section 2.1).

However, the increased production of insulin and its IAPP by-product by beta cells hyperactive mTORC1 may result in unfolded protein response (UPR), aimed at counteracting excess synthesis by suppressing protein synthesis and/or eliminating surplus. Elimination of surplus protein is accomplished by autophagy/lysosomal and/or proteasomal degradation. These degradation outlets are blocked by hyperactive mTORC1 [128], resulting in progressive ER stress followed by apoptosis [129]. Also, disruption of beta cells IR-Akt-FOXO1 pathway by hyperactive mTORC1 results in suppressing PDX and beta cells survival [130]. Hence, hyper activation of beta cells mTORC1 during the pre-diabetes phase of T2D serves as double-edged driver, allowing for beta cells high performance, while concomitantly promoting ER stress and beta cells apoptosis [131]. These two concomitant contrasting aspects of hyperactive mTORC1 may dynamically evolve during the clinical sequel of T2D, whereby the hyperplastic-hypertrophic initial feature yields progressively to an apoptosis outcome [131,132]. Of note, the double-edged profile driven by hyperactive mTORC1 in beta cells throughout the pre-diabetes and diabetes phases of T2D, questions the rational of drawing a pathological/etiological demarcation border between the two T2D stages.

#### Diabesity

90% of adult T2D patients are overweight or obese [4]. Increased bodyweight, and in particular visceral fat, is usually considered to be etiological in inducing insulin resistance and T2D, due to increase in adipose inflammatory agents (e.g. M1 macrophages, TNFa, IL6) and decrease in adipose adiponectin [133]. Others have argued for a protective effect of adiposity due to bypassing fat deposition in liver and muscle. The mTORC1-centric paradigm proposed here considers the obesity aspect of T2D to be a primary reflection of whole body hyper activation of mTORC1. The obesity disease is proposed to be induced and promoted by primary hyperactive hypothalamic mTORC1, resulting in disrupting the hypothalamic IR-Akt-FOXO1 transduction pathway which positively and negatively controls hypothalamic POMC/CART and NPY/AgRP, respectively [134, 135]. This will result in an orexigenic drive, suppression of energy expenditure, and disruption of net caloric balance. Increase in bodyweight gain is proposed to be further promoted by adipose hyperactive mTORC1/S6K1, resulting in PPARg-induced adipogenesis [136], SREBP-induced lipogenesis [137, 138] and suppression of ATGL-induced adipose lipolysis [139].

#### NAFLD

Most (~60%) T2D patients present with NAFLD [10]. Hepatic steatosis is due to active lipogenesis and suppression of LCFA oxidation. Lipogenesis and fatty acid esterification are transcriptionally controlled by SREBP, being activated by CRTC2 phosphorylation by hyperactive mTORC1 [137, 138]. Hepatic steatosis is proposed to be further promoted due to suppression of PPARa-induced beta-oxidation by hyperactive mTORC1 [140]. Hence, rather than presenting an insulin paradox (Section 2.2), the NAFLD / steatosis aspect of T2D reflects the double-edged activity of mTORC1 in promoting non-glycemic diseases of T2D while interfering with glycemic control. mTORC1 involvement in promoting liver fibrosis and cancerous transformation may account for the further progression of NAFLD to the cirrhosis and HCC stages, respectively.

#### Dyslipidemia

Most (60–90%) T2D patients are dyslipidemic [6–8]. T2D dyslipidemia triad (hypertriglyceridemia, increase in sdLDL-C, decrease in HDL-C) is driven by increase in VLDL-triglycerides (VLDL-TG) [141]. Increase in VLDL-TG is driven by hyperactive mTORC1 due to promoting hepatic steatosis, phosphatidylcholine synthesis [142], and disruption of the IR-Akt-FOXO1 pathway. FoxO1 activation results in transcriptional activation of the microsomal TG transfer protein (MTP) [143] and apoCIII [144]. MTP combines the VLDL ingredients to form the lipoprotein particle, and apoCIII suppresses plasma VLDL-TG lipolysis by lipoprotein lipase [141]. Of note, T2D dyslipidemia is avoided upon knocking out the IR [59], implying an obligatory role for insulin in promoting T2D dyslipidemia [60].

#### Hypertension

Most (60–85%) T2D patients are hypertensive [9]. Moreover, non-diabetic, non-obese hypertensive subjects present deranged glycemic control [145], indicating a putative common driver for T2D and essential hypertension. Indeed, hyperactive mTORC1 controls the three elements that determine blood pressure, namely, blood volume, vascular sympathetic tone and cardiac function. Fluid volume is controlled by renal sodium reabsorption carried out in the proximal and collecting duct by the apical passive Na/H NHE3 and ENaC transporters, respectively [146]. The apical transport flux is driven by basolateral Na/K ATPase [147], being controlled by the IR-Erk/RSK-transduced hyperactive mTORC1 [148–150]. Hypothalamic mTORC1 controls sympathetic tone [151], implying an increase in vascular tone and cardiac output. Increase in sympathetic vascular tone is further aggravated by vasoconstriction due to endothelial dysfunction (below). Chronic hypertension induces mTORC1-driven myocardial hypertrophy with increase in cardiac function, resulting eventually in heart failure. Hence, hyperactive mTORC1 may control the multiple drivers of T2D hypertension.

#### Macrovascular disease

The macrovascular disease accounts for half of all deaths of T2D patients [13]. The macrovascular disease is driven by two independent pathologic processes, namely, atherosclerotic cardiovascular disease (ASCVD) [152] and diabetic cardiomyopathy [153].

ASCVD is driven by endothelial dysfunction, resulting in infiltration and retention of sub-intimal VLDL/sdLDL remnants, followed by sub-intimal recruitment of monocytes / macrophages. Inflammatory cytokines (e.g., IL6, CRP, TNFa) produced by activated macrophages induce proliferation of vascular smooth muscle cells (VSMC), their attraction to the sub-intimal layer and their transformation into collagen secreting cells. Disruption of the fibrous cap of the lipid-fibrous plaque by macrophages elastase, metalloproteinase and collagenase results in thrombus formation and coronary occlusion [152, 154]. Hence, the ASCVD aspect of T2D is driven by diabetic dyslipidemia combined with diabetic endothelial dysfunction. Endothelial dysfunction is due to suppression of eNOS due to disruption of the IR-Akt pathway by hyperactive mTORC1 [155].

Diabetic cardiomyopathy is characterized by cardiac dysfunction and heart failure, not accounted for by coronary artery disease or hypertension [156]. It is driven by early left ventricle hypertrophy followed by systolic/diastolic dysfunction, reduced ejection fraction and increase in myocardial fibrosis and apoptosis. Diabetic cardiomyopathy may be improved by promoting cardiac autophagy or by rapamycin [157], implying a putative pathogenic role of hyperactive mTORC1. Hence, the macrovascular disease of T2D is proposed to be orchestrated by insulin-induced hyperactive mTORC1.

#### Microvascular diseases: Nephropathy, retinopathy, neuropathy

**Diabetic nephropathy** is characterized by detachment of glomerular podocytes from the epithelial basement membrane followed by their loss, and loss of proximal tubule cells. The combined loss results in albuminuria due to podocytes failure to filter out plasma albumin, and failure of proximal tubule cells to reuptake urinary albumin. Diabetic nephropathy may be delayed / prevented by IR knockout [158], or rapamycin treatment [159], or curtailing podocytes’ mTORC1 copies [160, 161], or by suppressing Akt activity in proximal tubular cells [162], implying a role for insulin-activated mTORC1 in driving diabetic nephropathy.

**Diabetic retinopathy** consists of non-symptomatic non-proliferative first stage retinopathy, followed by second stage proliferative neovascularization. The proliferative stage consists of extensive angiogenesis of leakage-prone blood vessels [163] driven by HIF1a-induced VEGF. HIF1a may be induced under normoxic conditions by hyperactive mTORC1 [164, 165].

**Diabetic peripheral neuropathy** is driven by decrease in density of small un-myelinated or thinly-myelinated intra epidermal nerve fibers (IENF) which originate in the dorsal nerve root ganglia and mediate pain, temperature sensation or autonomic functions. Hyperactive mTORC1 is reported to interfere with synaptic plasticity and to induce chronic neuropathy [166, 167]. In line with that, suppression of mTORC1 activity is reported to result in anti-nociceptive effects in experimental models of inflammatory and neuropathic pain [166–168].

#### Comorbidities

T2D is associated with increased risk of cancers (PDAC, CRC, breast, other), neurodegeneration (AD, Parkinson), psoriasis, poly cystic ovary syndrome (PCOS), other. Hyperactive mTORC1 is reported to drive these diseases, implying an etiological pathogenic rational for the association between T2D and the concerned comorbidities [169].

## Type 2 diabetes – Implications

### mTORC1-centric view of T2D

mTORC1 plays an important role in modulating and regulating metabolism across life cycle stages. Active mTORC1 is obligatory in controlling growth and development during the early age of organ development [97]. Hence, blocking its activity or maintaining its constitutive activity during the early age may result in pathology and disease. However, genetic, epigenetic or environmental conditions that maintain mTORC1 at that level of activity later in life may contribute to the development of T2D and its comorbidities. Hence, chronic dietary carbohydrate excess of high glycemic index, resulting in concomitant activation of the glucose-induced RagA/B.GTP and the insulin-induced Rheb.GTP drivers of mTORC1, may sustain mTORC1 hyper activity, and contribute to the T2D epidemic of modern times. Also, sedentary life style that avoids metabolic stress may further contribute to hyperactive mTORC1 [107–110] and the T2D epidemic. Indeed, suppression of mTORC1 activity in adult animal models is reported to delay and alleviate diseases of ageing and to increase lifespan [170, 171].

mTORC1 controls growth and metabolism in response to nutrients, energy and redox status, and its activity may be affected by genetic and/or epigenetic and/or tissue and/or context-dependent factors. Also, primary metabolic effects due to hyperactive mTORC1 may further be modulated by downstream secondary outcomes. Hence, an mTORC1-centric view of T2D conforms to the multifactorial complexity of T2D. However, this view does not exclude additional mTORC1-independent drivers that may complement hyperactive mTORC1 in phenotyping T2D and its associated comorbidities (cancer, neurodegeneration, PCOS, other).

### mTORC1-centric vs Gluco-centric paradigm of T2D

**Fig. 6:**
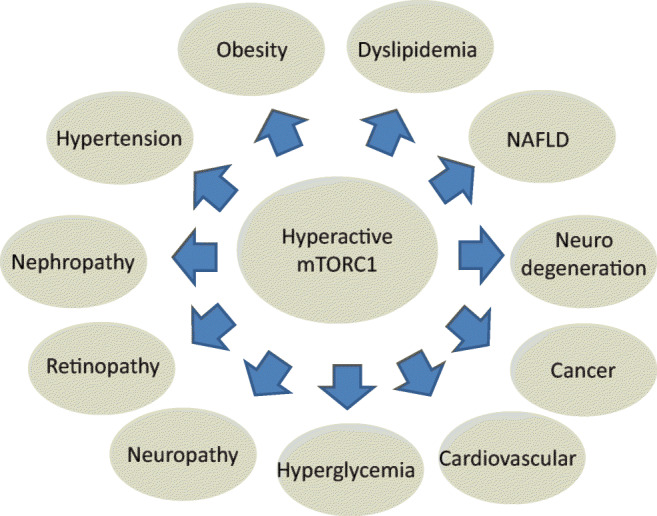
mTORC1-centric paradigm of T2D. The glycemic and non-glycemic disease aspects of T2D are driven by hyperactive mTORC1

The gluco-centric paradigm of T2D has provided an important and productive research, but also has limited the appreciation of other critical features involved in the pathogenesis and pathophysiology of T2D. The focus on hyperglycemia was driven by epidemiological data and by recognizing the benefit of strict glycemic control in delaying diabetic retinopathy, nephropathy and neuropathy in T1D patients [172, 173]. However, extrapolating the T1D lesson to T2D patients proved to be only partially justified [174] in view of the clinical reality of T2D [36]. Most importantly, the gluco-centric approach to T2D considers the non-glycemic diseases of T2D (obesity, dyslipidemia, hypertension, diabetic macrovascular disease) as ‘risk factors’ or secondary ‘comorbidities’ or ‘outcomes’ to be treated symptomatically (Fig. 1), rather than inherent primary aspects of T2D pathogenic context. Also, this view fails to realize the potential double-edged role of insulin in treating hyperglycemia while driving the non-glycemic diseases of T2D. The failure to recognize the exhaustive pathogenic context of T2D and the double-edged role of insulin therapy may have contributed to T2D being still an unmet need. In contrast, the mTORC1-centric paradigm considers hyperactive mTORC1 to be a primary driver and primary target for treatment for both, the glycemic and non-glycemic disease aspects of T2D (Fig. 6). Of note, secondary outcomes of hyperactive mTORC1, e.g., hyperglycemia, hypertension, dyslipidemia, glucolipotoxicity, oxidative stress, other, may further contribute to the T2D phenotype. Hence, the benefit of treating secondary outcomes is well appreciated. However, targeting secondary outcomes per se, while failing to target hyperactive mTORC1, should not be expected to disrupt the overall clinical progression of T2D.

### Pre-diabetes / diabetes

The pre-diabetes / diabetes paradigm of T2D maintains an inherent difference between the two stages of T2D. However, insulin resistance in the glycemic context, the non-glycemic diseases of T2D, and characteristics of beta cells failure are already evident during the pre-diabetes stage of T2D, implying a pathogenic continuum throughout the T2D disease. Hence, the pre-diabetes / diabetes paradigm of T2D does not conform to the pathogenic reality of T2D.

### T2D and insulin resistance

‘Insulin resistance’ is considered to be a cornerstone of T2D pathogenesis. However, ‘insulin resistance’ may only refer to the glycemic context of T2D, whereas the non-glycemic aspects of T2D are fully responsive to insulin and driven by hyperinsulinemia. Since the non-glycemic diseases of T2D are of major role in profiling T2D morbidity and mortality, the canonical view of ‘insulin resistance’ misses the exhaustive pathogenic scope of T2D. In contrast, the ‘mTORC1-centric’ paradigm may better describe the double-face of resistance and response to insulin in the T2D context (Fig. 5). Moreover, in terms of molecular targets for treatment, ‘hyper active mTORC1’ may offer an explicit target for an ‘all-in-one’ treatment of T2D. That is in contrast to ‘insulin resistance’ which indicates a phenotypic feature rather than a specific molecular target.

### Insulin sensitizers

**‘**Insulin sensitizers’ (e.g., metformin, glitazones) are considered to be agents that promote response to insulin. While this is correct in relating to the glycemic context, these agents suppress the effects of insulin on the non-glycemic diseases of T2D. Indeed, ‘insulin sensitizers’ are all inhibitors of mitochondrial complex I [84, 175], resulting in suppressing mitochondrial electron flux and oxidative phosphorylation, with increase in metabolic stress and AMPK [176, 177]. Metabolic stress and AMPK inhibit mTORC1 activity (Section 3.1), thereby enhancing the glycemic effects of insulin (Section 3.3), while suppressing insulin action in driving the non-glycemic diseases of T2D (Section 3.4). Hence, the canonical view of ‘insulin sensitizers’ fails to realize their double-edged feature and their potential therapeutic benefit in counteracting and alleviating the non-glycemic diseases of T2D during the sub-hyperglycemic pre-diabetes stage.

### Insulin(s)

Insulin therapy, by either promoting its endogenous production and secretion while beta cells are still functional, or by its exogenous supply upon beta cells failure, is a corner stone of current T2D treatment. However, since insulin drives mTORC1 hyper activation, and since hyperactive mTORC1 disrupts the IR-Akt transduction pathway, insulin may serve as potent driver in promoting resistance to insulin in the glycemic context. Hence, insulin presents a double-edged agent, being required for controlling hyperglycemia while concomitantly promoting resistance to insulin in the glycemic context [178]. Indeed, insulinoma patients are resistant to insulin and recover to normal sensitivity upon tumor resection [179]. In line with that, the percentage of T2D patients who reach an HbA1C target <7.0% progressively decrease with advancing anti-diabetic treatments, amounting to 88% and 36% in patients treated with metformin only or with a variety of antidiabetic drugs including insulin, respectively [27]. Moreover, chronic insulin treatment may drive the non-glycemic diseases of T2D due to mTORC1 hyper activation via the IR-Erk/RSK transduction pathway. The apparent improvement of beta cells function by short-term intensive insulin treatment in early T2D patients [180] may delay, but not prevent, the progressive beta cells failure driven by chronic insulin treatment and hyperactive mTORC1 [131]. In light of the major role played by the non-glycemic diseases of T2D in profiling T2D morbidity and mortality as well as in promoting T2D comorbidities (e.g., cancer, neurodegeneration, other), chronic insulin doses should be carefully considered beyond its risk of hypoglycemia. In line with that, non-insulin therapies (e.g. metformin, SGLT2i) that may reduce reliance on insulin as a mainstay for treatment should be prioritized.

### Prospective treatment approaches

Suppression of hyperactive mTORC1 may offer an all-in-one treatment for T2D in terms of pathogenesis, clinical focus and treatment strategy. Indeed, suppression of hyperactive mTORC1 may allow for the IR-Akt pathway to become responsive to insulin while avoiding the non-glycemic diseases of T2D. Suppression of hyperactive mTORC1 may further be expected to result in beta cells rest and preserving their function. mTORC1 may be targeted by caloric restriction or by carbohydrate restriction, resulting in suppressing the Rheb.GTP and RagA/B.GTP drivers of mTORC1. Ketogenic diets may indeed offer carbohydrate restriction, but at the expense of activating mTORC1 by dietary amino acids [181]. However, the compliance to caloric restriction and/or carbohydrate restriction and/or ketogenic diets is poor. Also, caloric restriction induced by bariatric surgery has limited relevance for the expanding T2D population. Treatment of T2D patients with rapalogs is dubious in light of their side effects [182], and since chronic treatment may result in inhibition of mTORC2, thereby suppressing the IR-Akt transduction pathway. The long-term success of intermittent treatment with rapalogs [183] still remains to be fully evaluated. Of note, the recently reported partial efficacy of GLP1 analogs and SGLT2i in alleviating the macrovascular disease of T2D may putatively be ascribed to their suppression of mTORC1 hyperactivity due to negative energy balance (GLP1 [184]) or urinary excretion of glucose calories (SGLT2i [185]), rather than their mild hypoglycemic efficacy. The mitochondrial complex I / mTORC1 connection exemplified by metformin [175,186] may prompt a search for novel complex I inhibitors designed to suppress mTORC1 specifically.
